# SAXS/WAXS Investigation of Amyloid-β(16-22) Peptide Nanotubes

**DOI:** 10.3389/fbioe.2021.654349

**Published:** 2021-03-24

**Authors:** Theyencheri Narayanan, Axel Rüter, Ulf Olsson

**Affiliations:** ^1^ESRF-The European Synchrotron, Grenoble, France; ^2^Division of Physical Chemistry, Lund University, Lund, Sweden

**Keywords:** peptide self-assembly, peptide nanotubes, peptide nanoribbons, X-ray scattering, SAXS

## Abstract

This brief report presents an X-ray scattering investigation of self-assembled nanotubes formed by a short peptide. X-ray scattering methods enable multiscale structural elucidation of these nanotubes in solution under the same conditions involved in the self-assembly process. In particular, the dimensions of nanotubes and the crystalline organization within their walls can be determined quantitatively. This is illustrated in the case of amyloid-β(16-22) peptide nanotubes.

## 1. Introduction

The hierarchical self-assembly of short peptides to form well-defined nanotubes of nearly macroscopic dimension has been the subject of numerous investigations (Childers et al., 2009; Valéry et al., 2011; Hamley, 2014). Most often short peptides self-assemble to form fibrillar morphologies but under certain specific conditions the ribbon-like fibrillar structure curls and form nanotubes of uniform dimension. This self-assembly is governed by the delicate interplay of hydrogen bonding, electrostatic, and entropic interactions. Both helical ribbons and nanotubes have similar mean curvature and zero Gaussian curvature (Ke et al., 2020). The size uniformity and tunability make them suitable for templated growth of functional nanomaterials with potential applications in modern technologies (Valéry et al., 2011; Hamley, 2014; Levin et al., 2020). The preceding article presented a short review of X-ray scattering investigations of different peptide systems forming similar nanotubes (Narayanan et al., 2021).

A well-known example for the nanotube forming peptide system is the *CH*_3_*CO* − *KLVFFAE* − *NH*_2_, a sequence from the amyloid-β peptide [Aβ(16-22)] (Lu et al., 2003; Mehta et al., 2008). This short peptide self-assembles in acetonitrile/water binary liquid mixture at pH 2 forming well-defined nanotubes with mean diameter and wall thickness about 52 and 4.3 nm, respectively. This system has been the subject of many structural investigations (Childers et al., 2009). Instead at pH 6, this peptide exhibits fibrillar morphology (Mehta et al., 2008). Using electron diffraction and complementary spectroscopic methods, the packing of peptides into bilayer leaflets within the tube wall was demonstrated (Mehta et al., 2008, 2013). A model for the lamination of peptides involving antiparallel β-sheets and the curling of peptide bilayers to form nanotubes was proposed (Childers et al., 2009).

This report presents a small and wide angle X-ray scattering (SAXS and WAXS, respectively) investigation of the Aβ(16-22) self-assembly over a wider peptide concentration range. A combination of SAXS and WAXS methods elucidates the different hierarchical levels exhibiting by the self-assembled structure. The improvement in the detection capability of scattering techniques now enable deciphering weak structural features submerged beneath a high background as illustrated here. In addition, a coexistence of two different structural moieties is observed at higher peptide concentrations.

## 2. Materials and Methods

The Aβ(16-22) peptide (*CH*_3_*CO* − *KLVFFAE* − *NH*_2_) nanotubes were formed in 40 weight % acetonitrile/water solvent mixture with 0.1% trifluoroacetic acid (TFA) at pH close to 2 (Lu et al., 2003; Mehta et al., 2008). The peptide was purchased from CPC Scientific Inc. (purity of 97 %) and was used without further purification. The pH of the solvent was adjusted to 2 by the addition of TFA. Nanotubes were aged for several weeks prior to SAXS and WAXS measurements. The X-ray scattering experiments were performed on the ID02 beamline at the ESRF (Narayanan, 2014). The samples were loaded in a flow-through capillary cell (diameter ~ 2 mm and wall thickness ~ 10 μm) maintained at 25^*o*^C, which enabled accurate background measurements from the capillary filled with the solvent as well as limited flow alignment of the nanotubes. To cover the broad size scales relevant to the self-assembled structure, two sample-to-detector distances of 8 and 1.2 m were used for SAXS and the WAXS detector was placed at 0.11 m from the sample. The measured two dimensional scattering patterns were normalized to an absolute intensity scale after applying detector specific corrections and isotropic patterns were azimuthally averaged to obtain the one-dimensional scattering profiles (Narayanan, 2014). After subtraction of the corresponding normalized background, one-dimensional profiles from different sample-to-detector distances were merged together to obtain the scattered intensity, *I*(*q*) as a function of the magnitude of scattering vector (*q*). Where *q* = (4π/λ)*sin*(θ/2), with λ the wavelength of the X-rays (≃ 1 Å) and θ the scattering angle. The SAXS data were modeled in terms of core-shell cylinders (with the core scattering length density set to the same as the solvent) and rectangular parallelepiped form factors using the SasView software (Doucet et al., 2017). The helical ribbon form factor was adapted from the models presented in Pringle and Schmidt (1971) and Hamley (2008).

## 3. Results and Discussion

**Figure 1:**
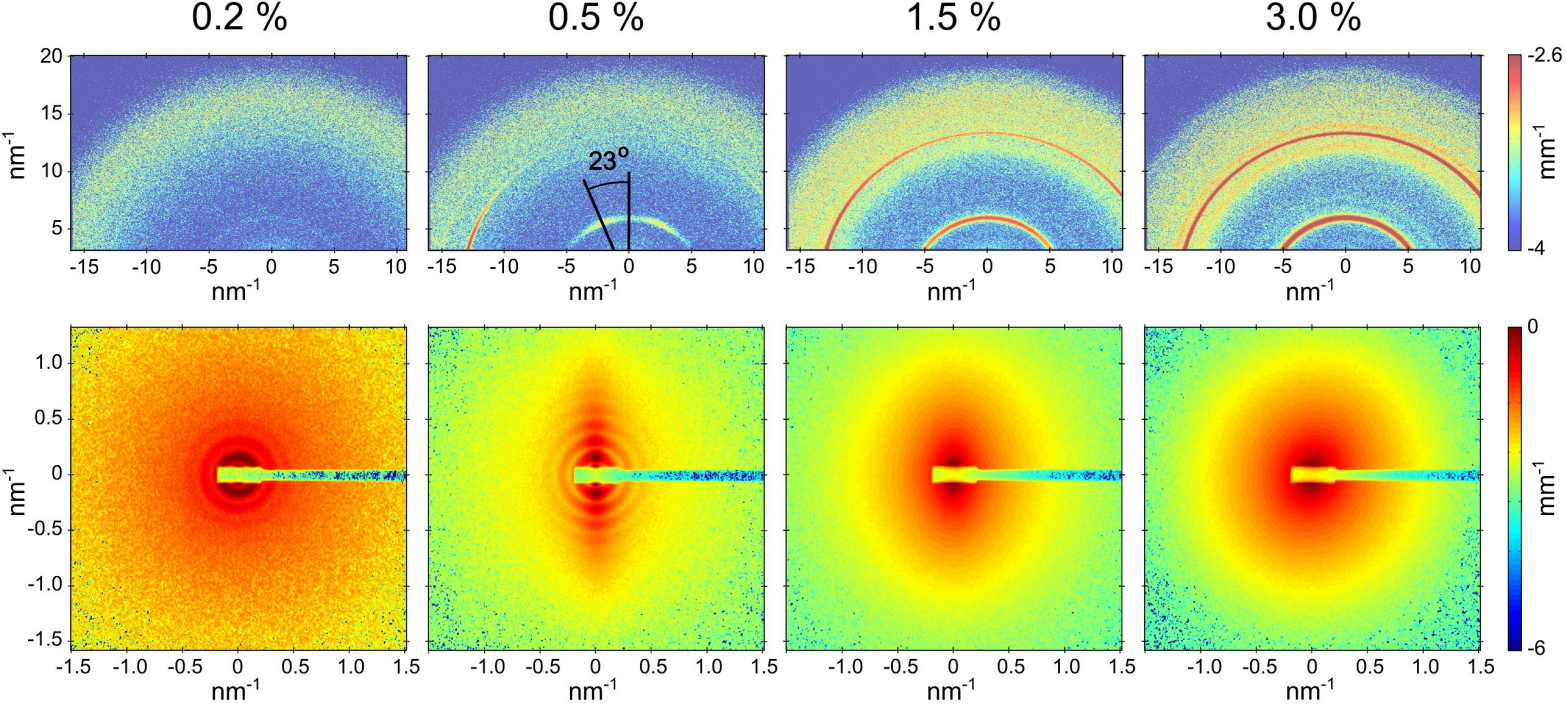
Partially aligned SAXS and WAXS patterns (lower and upper panels, respectively) from Aβ(16-22) peptide samples at four different concentrations after the solvent background subtraction. In the case of 0.5% sample, the nanotubes are aligned along the horizontal axis and the relative orientation of nanotube radius and β-sheet laminations is shown. The SAXS sample-detector distance was 1.2 m.

Figure 1 presents the partially oriented 2D SAXS and WAXS patterns for four concentrations of the peptide over a restricted *q* range. At 0.1 % (by weight), the peptide was fully soluble without any features of nanotube scattering function. The 0.2 and 0.5% samples clearly showed the scattering form factor of nanotubes as indicated by the oscillations in SAXS intensity and corresponding WAXS patterns displayed weak Bragg peaks from the peptide packing within the nanotube walls. The 0.5% sample maintained the alignment of nanotubes induced by the flow while in 0.2% sample the orientation was lost upon cessation of the flow. The clear alignment of WAXS peaks in the 0.5% sample indicates that the β-sheets are oriented around the nanotube axis along a helical path with a pitch angle of 23^*o*^. A distinguishing hallmark between the tubular and fibrillar morphologies is this azimuthal tilt in the β-sheet reflections. The SAXS patterns from 1.5 and 3.0% samples did not show the characteristic features of nanotube scattering form factor though some anisotropy is maintained in the former. Corresponding WAXS patterns displayed stronger Bragg peaks without clear azimuthal orientation. The observed SAXS features suggest a morphological transition but WAXS confirms that the molecular packing of peptides has not changed. This peptide is known to form nanotubes only around pH 2, whereas at higher pH a fibrillar assembly has been observed (Mehta et al., 2008). Here, the pH is fixed at 2 by the presence of the strong acid TFA, and the lysine side group remains fully protonated at all concentrations studied.

**Figure 2:**
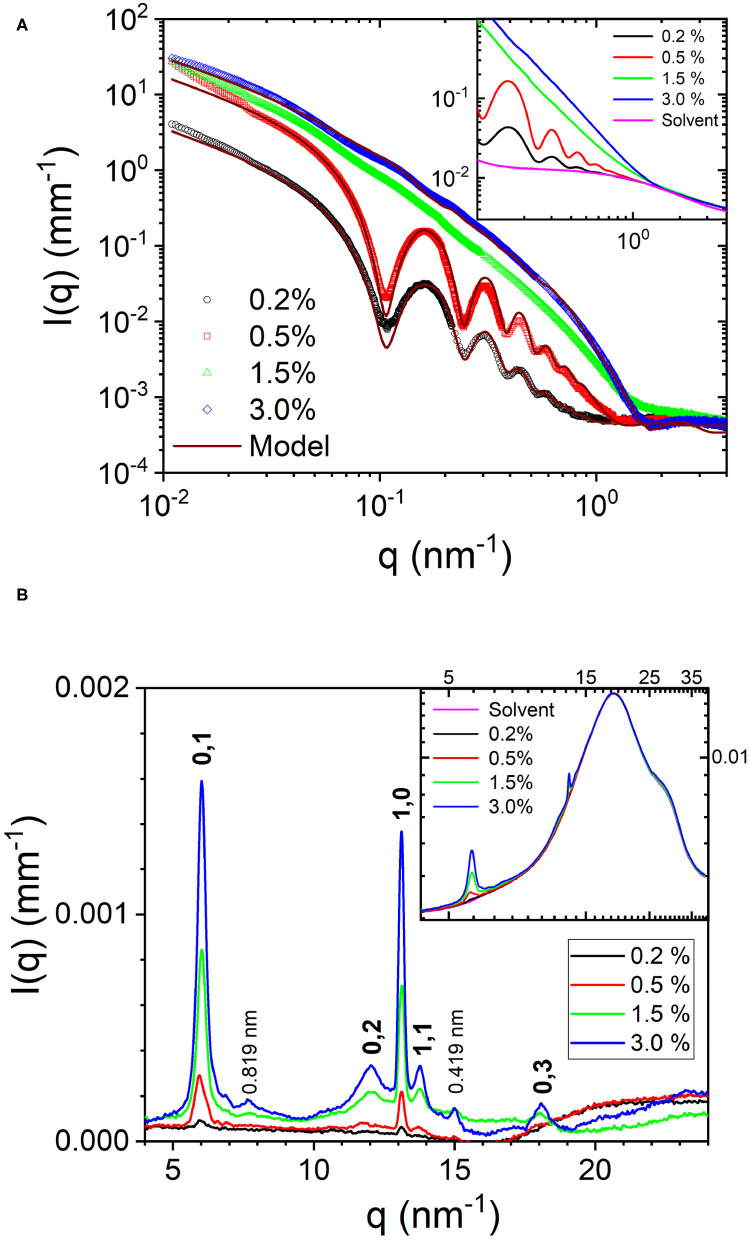
**(A)** Normalized SAXS profiles from Aβ(16-22) peptide at four concentrations over the full *q* range of measurement. The inset displays the background unsubtracted profiles and highlights the importance of accurate background subtraction. **(B)** Corresponding WAXS profiles for the four peptide concentrations and indexation of the Bragg peaks (the spacings of which are specified in the text). The inset depicts the strong solvent background scattering.

Figures 2A,B depict the azimuthal average of the normalized SAXS and WAXS intensities, respectively over the full *q* range of measurements. To derive the structural parameters of the nanotubes, SAXS profiles were modeled by polydisperse core-shell (hollow) cylinder scattering function in SASView. The corresponding parameters, outer radius (*R*) and wall thickness (δ), were 24.8 and 5.2 nm, respectively for 0.2% sample and 24.6 and 4.4 nm, respectively for 0.5% sample. The mean length of the nanotubes was larger than the *q* range covered by these measurements. In the fits, it was kept in the range of 3,000–5,000 nm but from the alignment behavior, it is clear that the nanotubes are much longer. The obtained size parameters are consistent with previously reported values (Lu et al., 2003; Mehta et al., 2008). In the modeling, the wall thickness is constrained by the scattering minimum around *q* of 1–2 nm^−1^, which was not resolved in previous studies. The inset of Figure 2A shows that the determination of this high *q* minimum critically depends on the accuracy of the solvent background subtraction. Due to concentration fluctuations, the acetonitrile/water solvent mixture background is a Lorentzian function (Narayanan, 2014) and not flat like that of pure water. The high *q* minimum of the nanotube scattering falls on the decaying part of the Lorentzian function with a correlation length ≃ 0.8 nm. Nevertheless, the shift in the minimum is evident even in the background unsubtracted data. Although the outer diameter is nearly the same, the packing of peptides in the 0.5% sample appears to be tighter. The SAXS profiles of 1.5 and 3.0% samples showed only weak oscillations and the minima and maxima are less defined. This suggests a coexistence of two morphologies, which are likely nanotubes and nanofibrils. As a result, SAXS profiles were modeled by a linear combination of polydisperse hollow cylinder and monodisperse parallelepiped scattering functions. In addition, the weak minima and maxima have shifted to lower *q* values than in the 0.5% case implying an increase in the diameter of nanotubes or partial unwinding (helical ribbons). The model curve for 3.0% sample represents nanotube radius and wall thickness of 33.6 and 4.0 nm, respectively, and parallelepiped length, width, and thickness of 3,900, 36.0, and 3.5 nm, respectively. The corresponding nanotube and nanofibril number density fractions are 0.23 and 0.77, respectively. Table 1 summarizes the main structural parameters derived from the SAXS modeling in terms of a linear combination of tube (hollow cylinder) and flat ribbon (parallelepiped) scattering functions.

**Table 1:** Model parameters from the SAXS analysis of Aβ(16-22) peptide samples shown in Figure 2A.

**Concentration**	**Mean radius**	**Wall thickness**	**Polydispersity**	**Fraction**	**Length**	**Width**	**Thickness**
**(weight %)**	**(nm)**	**(nm)**			**(nm)**	**(nm)**	**(nm)**
0.2	24.8	5.2	0.09	1.0			
0.5	24.6	4.4	0.07	1.0			
1.5	34.0	4.0	0.06	0.63	3,900	36.0	3.5
3.0	34.0	4.0	0.06	0.23	3,900	36.0	3.5

*Columns 2-5 correspond to nanotubes and the remaining (6-8) for nanofibrils. The number density fraction is a relative quantity with total nanotube and nanofibril fractions summing to 1. The error bars are in the last significant digit*.

For the 1.5 and 3.0% samples, the SAXS profiles can also be described by a linear combination of helical and flat ribbons with parameters of helix, mean radius, polydispersity, width, and length; 62, 0.06, 80, and 2,000 nm, respectively. The pitch was fixed at the same value as that of the tube (364 nm), determined by its radius and angle from the WAXS pattern (67^*o*^). The helical ribbon form factor alone is not sufficient to describe the SAXS profiles and a clear power law decay of *I*(*q*) at the high *q* region is absent (Hamley, 2008). The lack of significant orientation suggests that the nanotubes and nanofibrils are shorter than the nanotubes in the 0.5% sample. This is also consistent with the tendency of scattering profiles to flatten at lower *q* region. The structure factor of interparticle interactions (Narayanan, 2014) is neglected as the data do not display the signature of such an effect in 1.5 and 3.0% samples. Moreover, for a given peptide volume fraction (ϕ_*p*_), the hollow nanotubes occupy a larger volume than ribbons as they also contain enclosed solvent. The nanotube volume fraction, ϕ_*tube*_, is approximately given by ϕ_*tube*_ = (*R*/2δ)ϕ_*p*_ (Bucak et al., 2009). For the parameters in Table 1, nanotubes are roughly 3 times more voluminous than flat ribbons for the same ϕ_*p*_.

**Figure 3:**
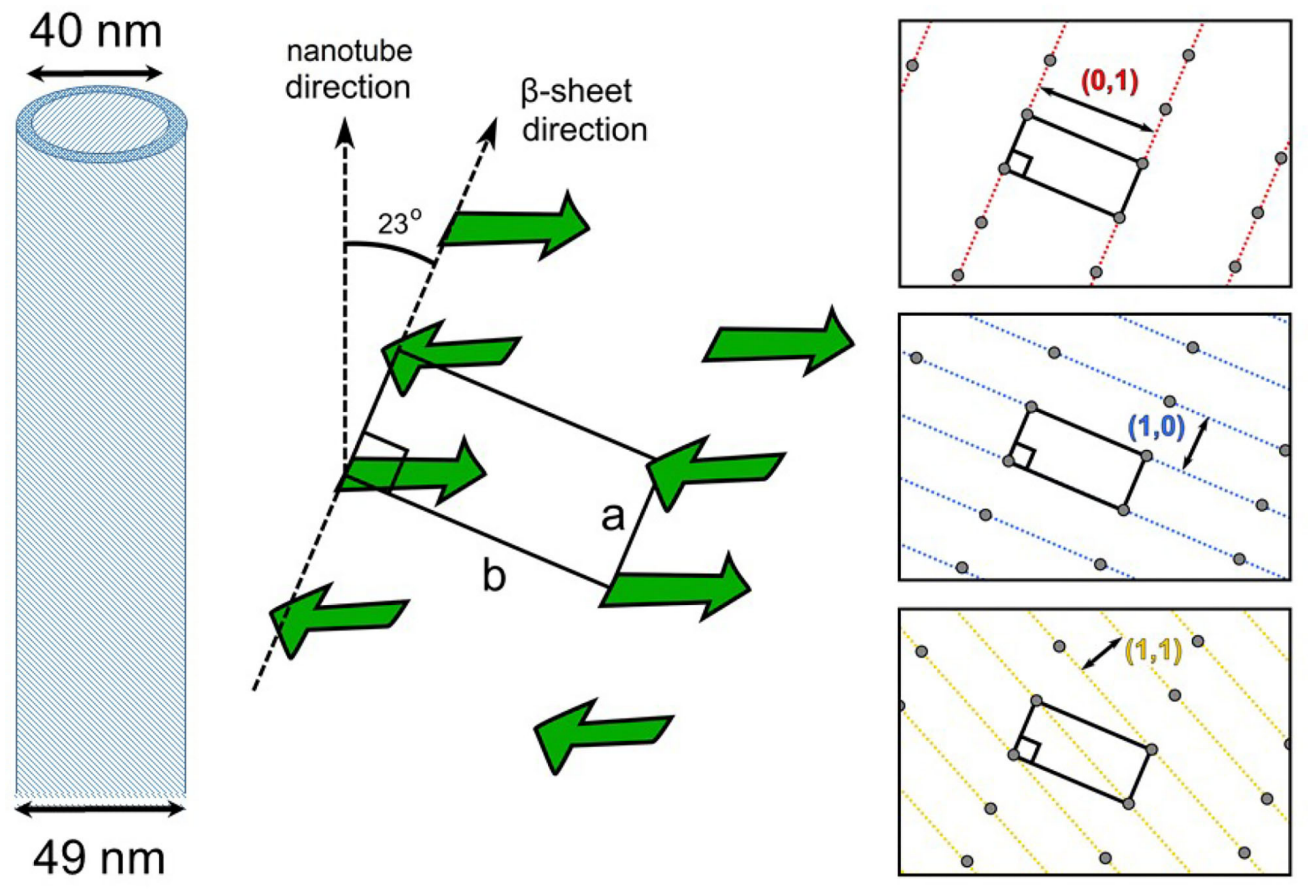
Schematic illustrations of the nanotube, the semi-crystalline architecture within the walls and corresponding orientation of 2D lattice planes of a rectangular unit cell with side lengths a = 0.479 nm and b = 1.046 nm.

The azimuthally averaged WAXS profiles are shown in Figure 2B. The inset displays the corresponding background unsubtracted data and shows the weak Bragg peaks superimposed on the strong structure factor of the solvent mixture. In addition, the perfect overlap of the solvent scattering confirms that the composition of the solvent remained the same in all four samples. The stronger Bragg peaks at 13.1 and 6.0 nm^−1^ corresponding to spacings of 0.479 and 1.046 nm represent the orthogonal β-strand periodic repeat and stacking of the antiparallel β-sheets, respectively. Interestingly, both these periodicities are very similar to the two orthogonal cross-β spacings, meridional and equatorial, respectively, observed in the fiber diffraction diagram of full length Aβ peptide (Serpell, 2000). These Bragg peaks can be assigned to the (1,0) and (0,1) reflections from a 2D rectangular lattice (Childers et al., 2009), schematically shown in Figure 3. The higher order peaks of the stacking or lamination can be found at 12 and 18 nm^−1^, corresponding to spacings of 0.523 and 0.348 nm, respectively. In the case of nanofibrils, two additional weak peaks at 7.7 and 15 nm^−1^ (spacings 0.819 and 0.419 nm) are also observed, which may be from out of registry β strands. The observed peak positions in the WAXS are consistent with that reported by electron diffraction (Mehta et al., 2008), except for 5–10% dilation of spacings, which can be attributed to the drying effect in the latter. The advantage of WAXS is that the information is deduced in the solution condition that is more relevant in functional studies. Figure 3 schematically illustrates the semi-crystalline organization within the nanotube walls and different crystallographic planes of the 2D lattice.

A transition from nanotubes to helical ribbons and then to nanofibrils has been observed in the case of amphiphilic peptide *KI*_4_*K* with increasing acetonitrile in a similar aqueous solvent (Zhao et al., 2015). However, a large change in the relative concentration of acetonitrile (20–80 %) was required. These transformations have been attributed to the changes in the hydrophobic interaction in the side chains while the peptide backbone hydrogen bonding remained intact (Zhao et al., 2015). The amphiphilic peptide system *A*_6_*R* is another example, where a coexistence of nanotubes and nanosheets has been observed at higher peptide concentrations (> 15%) (Hamley et al., 2013). In that case, the transition is attributed to enhanced screening of the electrostatic repulsion between the arginine units and C-terminal carboxyls, which stabilizes the planar geometry. For the related peptide A_6_K a coexistence of nanotube and a high concentration planar lamellar phase has been reported (Cenker et al., 2011). The mechanism for the concentration induced structural transition from nanotubes to nanofibrils in the present case is not fully clear. The relative stabilities of nanotubes and twisted ribbons were recently discussed within a model considering different β-sheet deformations in the two structures in addition to a preferred β-sheet twist and an interfacial tension term (Rüter et al., 2020). The peptide chemical potential in the two structures can be very similar, and small perturbations (e.g., translational and rotational entropies) may shift the relative stability from nanotubes to nanofibrils.

## 4. Conclusion

Overall, the presented SAXS and WAXS data are in good agreement with the nanotube model derived from complementary electron microscopy and diffraction, an array of spectroscopic methods, and molecular dynamics simulations (Mehta et al., 2008, 2013; Childers et al., 2009). The nanotube diameter and wall thickness are consistent with the values reported earlier (Lu et al., 2003; Mehta et al., 2008) and compatible with an interdigitated bilayer arrangement of peptides in the nanotube walls. However, as the peptide concentration is increased, there is a gradual unwinding of the nanotubes, and partially unwound tubes or helical ribbons coexist with flat ribbons.

The example presented in the previous section illustrates the capability of scattering methods to decipher weak structural signals submerged beneath a large solvent background. This aspect could be further exploited in functional studies of self-assembled peptide systems. A combination of SAXS and WAXS methods enable hierarchical structural elucidation from the molecular packing up to the macroscopic dimension of the nanotubes simultaneously. In addition, the coexistence of different structural moieties can be identified on a quantitative scale.

## Data Availability Statement

The raw data supporting the conclusions of this article will be made available by the authors, without undue reservation.

## Author Contributions

UO and TN designed the research and TN performed X-ray measurements. TN and AR analyzed the data and all authors involved in the discussion of the results. TN wrote the first draft of the manuscript. All authors contributed in the revisions of the manuscript.

## Conflict of Interest

The authors declare that the research was conducted in the absence of any commercial or financial relationships that could be construed as a potential conflict of interest.
