# Supplementary figures and images for: SAXS/WAXS Investigation of Amyloid-β(16-22) Peptide Nanotubes
Source: Front Bioeng Biotechnol. 2021 Mar 24;9:654349. doi: 10.3389/fbioe.2021.654349 (PMC8024549; doi:10.3389/fbioe.2021.654349)

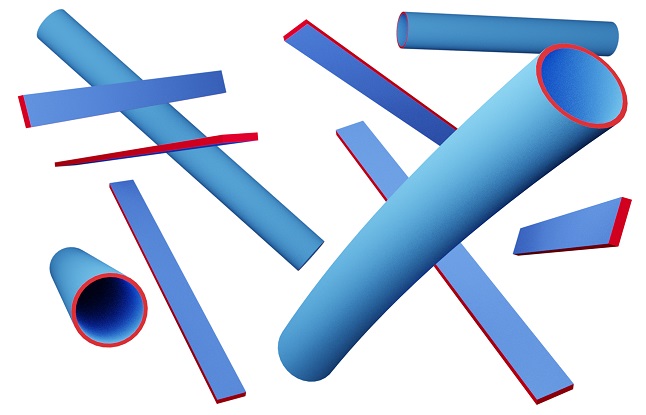

Supplement: Supplementary file 1 [file Image_1.JPEG]
